# Why regulation hurts: balancing the need to maintain standards with the mental health impact on public sector professionals

**DOI:** 10.1192/bjb.2024.90

**Published:** 2025-06

**Authors:** Hester Mannion, Darren Minshall, Mark Tarn, Derek K. Tracy, Samantha K. Brooks, Neil Greenberg

**Affiliations:** 1 East London Foundation Trust, London, UK; 2 UK Defence Medical Services, Plymouth, UK; 3 Guy's and St Thomas’ NHS Foundation Trust, London, UK; 4 West London NHS Trust, London, UK; 5 King's College London, London, UK

**Keywords:** Occupational psychiatry, occupational medicine, moral injury, suicide, public sector

## Abstract

Robust regulatory scrutiny is an unavoidable and necessary part of professional life for public sector professionals. Inspection and investigation can lead to poor mental health for individuals already working under pressure owing to increased workload and anticipation of poor outcomes. Although good regulation maintains standards and provides accountability to government and the public, regulators must face their obligation to understand the wider impact of their practices on the mental health of those they evaluate. This article discusses how regulation affects public sector culture and the potential risks and negative impact of regulatory practices and highlights how clinicians, working in occupational practice, are well placed to recognise ‘regulatory stress’ among public sector workers and offer vital support, guidance and advocacy.

Regulatory scrutiny is a complex combination of unavoidable, necessary and appropriate governance which is stressful for most professionals and their organisations. In the UK, public services often face intense regulation procedures compared with private and third-sector organisations.^1^ With limited government funding available and the need for safe standards for the most vulnerable to be upheld, demand for accountability to the public and their Parliament is unsurprising and indeed appropriate.

## Organisational regulation in the UK

The UK has a long history of regulating organisations, born out of the privatisation of nationalised industries throughout the 20th century. Today, more than 90 regulatory bodies are functioning in the UK, which is considered a world leader in good regulatory practice; the UK's experience of regulation serves a key role in global diplomacy.^1^ Regulatory reform has been targeted by successive UK governments since the 1990s, with a particular focus on value for money in the private sector. This was typified by the ‘one-in, two-out’ rule devised by the Cameron government, which required an overall reduction in the cost balance of new regulation in a bid to boost private sector productivity, maintaining standards without burdening business with excessive bureaucracy and inspection.^2^

Sectors such as education and healthcare, which are used by some of the most vulnerable groups and populations, should arguably face especially thorough regulation. Indeed, public services are subject not only to the law but also scrutiny by service-specific bodies and inspectorates. These organisations have a clear and vital role. Good regulation should provide accountability to Parliament and to regulated entities and, of course, confidence for the public. Effective regulatory bodies should produce guidance on practice, as well as conducting inspections and investigations, to maintain standards, justify the use of public funds and safeguard public interest.

There is no shortage of negative attention from the media when public services fall short of regulatory standards, but, until very recently, there has been a lack of demand for the regulators themselves to be accountable for their judgements. Furthermore, there has been little scrutiny of the potential negative impact of bad regulatory practices on the mental health and well-being of public sector workers and public sector culture more broadly. This may be partly due to the confusing and heterogenous organisational structure of the regulatory sector. Many bodies have evolved organically and range from non-departmental public bodies to executive agencies, non-ministerial departments and statutory corporations. It is not straightforward for insiders, and often confusing for outsiders, making it difficult to easily understand who is accountable to whom.

## The occupational hazards of regulation

In 2023, there was an unprecedented outpouring of public concern about Office for Standards in Education (OFSTED) inspection practices. This followed the suicide of primary school headteacher Ruth Perry; the coroner subsequently determined that a school inspection had contributed to her death.^3^ This has resulted in renewed discussion and debate about the roles and responsibilities of regulators, and in particular the duty of care to those being regulated. OFSTED has since been found wanting, with some inspection practices deemed as unnecessarily pressured, isolating and intimidating. OFSTED had no written policies regarding the management of school leader anxiety during the inspection process.^3^ The Leeds University Hazards Campaign seeks to have work-related suicides reported to the Health and Safety Executive and investigated on par with other work-related deaths.^4^ Their investigations identified a further ten incidents where OFSTED had been mentioned in coronial reports in the past 25 years. Without better data, it is difficult to determine whether regulation was linked to other such deaths.

Other professional regulators face similar scrutiny. The General Medical Council (GMC) reviewed the 114 deaths among doctors under investigation between 2005 and 2013 and found that 28 died from suicide or suspected suicide. This led to a range of recommendations for process reform.^5^ The GMC now produces an annual report of deaths for doctors under investigation, promoting transparency and accountability. In the 4 years between 2018 and 2020, the GMC reported the deaths of 29 doctors while under investigation or monitoring, with five confirmed cases of suicide.^6^

There is consensus as to what good regulation should look like; it should be proportional, accountable, consistent, transparent and targeted.^7^ However, despite good intent, several types of harm can result from regulatory inspection. At a service level, bureaucracy and excessive scrutiny can stifle creativity and lead to a diversion of energy away from the front line. The result can draw the clinician from the bedside, the teacher from the whiteboard or the officer from the beat. Regulation can also be a cause of ill health. Teachers report increased levels of sickness and burnout following inspections, with an overwhelmingly negative impact on their mental well-being. ^8,9^ We also know that public sector workers have higher rates of stress and poor mental health compared with their private sector counterparts.^10^ Several factors may contribute to this, and it is entirely possible that intrusive and disproportionate regulation can be implicated through increased workloads, poor morale and rising stress levels.^11–13^

For occupational health clinicians, a focused awareness of the risks of ‘regulatory stress’ and the associated mental health impact for highly regulated professionals is valuable. In their management standards for stress, the Health and Safety Executive identifies six key areas of work design that, unless properly managed, are associated with poor health and increased sickness absence.^14^ These include high workload demand, poor control over working practices and poor communication about change in the workplace, domains over which regulation can have a huge impact.

Public sector organisations also tend to be large and complex, and they are subject to external pressure and influence. Public sector workers at all levels have to operate in a frequently shifting landscape, complicated by changes to government policy and targets that may have more to do with political sensitivities than service improvement. These volatile, uncertain, complex and ambiguous environments can be especially difficult for public services to navigate.^15^ Professionals at different organisational levels will also experience regulatory pressure in unique ways; senior leaders will be accountable for organisational performance, whereas front-line practitioners have their delivery of everyday tasks scrutinised.

The close alignment of personal and professional identities is also of relevance. Workers with a high vocational drive are likely to be overrepresented in the public sector, especially in healthcare and teaching. These individuals are likely to have their occupational identity and sense of self-worth closely intertwined. There is evidence that strong vocational attitudes may be associated with greater resilience, better self-efficacy, higher quality of work and better integrated working.^16^ For some professionals with closely aligned identities, profession-specific regulatory bodies can have a positive impact, supporting, bolstering and legitimising professional status. However, for vocational individuals, having closely aligned personal and professional identities also increases their vulnerability, and so should it increase the duty of care from regulators. It is all too often the case that the relationship between the public sector professional and the regulatory body is riddled with defensiveness, conflict, and fear of heavy handedness or arbitrary judgement.^17^ Perceived disproportionate or unreasonable feedback by a regulator that fails to acknowledge external pressures such as chronic underfunding and staff shortages is deeply demoralising and may lead to embitterment.^18^ This could also compound moral distress felt by professionals already concerned about being too over-stretched to provide a good service.^19^

High-profile cases where health professionals, social workers, teachers and police officers have deliberately harmed or neglected vulnerable people understandably feeds public anxiety. This, in turn, increases the perceived need for reassurances that measures are being taken to minimise all possible risks. At a societal level, the idea that risk can be eliminated can feed in to ‘moral panic’ about a deterioration in public health, policing, social care and education standards.^20^ Without care, an impossible-to-achieve drive to remove all risk to service users can engender a demand for infallibility in professionals. Inevitable failure in this regard will, in turn, feed the desire for more regulation and fuel unhelpful hostility between professionals and their regulators.

## A call for regulatory practice obligations

The anxiety and despair expressed in Ruth Perry's own personal notes provides a rare and troubling insight into the devastating impact such pressures can have on mental health. Her case and numerous research studies into school communities undergoing inspections have shown that anticipation of judgement and the time in limbo before public revelation of a damning report, are especially damaging. The longer an investigation is drawn out, the more intense the pressure on an individual, the higher the risk to their mental health.^21^ Protracted investigations and judgements can lead to isolation from vital professional support groups. This is especially true if an individual is suspended as part of the investigation process and colleagues are instructed not to communicate with them. This highlights the vital importance of the mode and timing of feedback from regulators. Communication should be managed with care and shared consensually, transparently and, wherever possible, without nasty surprises.

Regulators must face their obligation to better understand the context of resource constraints and avoid blaming individuals where systemic or cultural factors are outside personal control. It is problematic to have regulators’ actions, even unintentionally, contributing to the recruitment and retention crisis that blights many UK public services. In view of their weighty influence, regulators’ success must be measured in more than targets to raise standards; they should be required to explicitly assess the benefit of such scrutiny against the risks of leaving a disillusioned, exhausted and possibly suicidal workforce in their wake.

Clinicians who work in occupational practice, supporting public sector workers, may recognise ‘regulatory stress’ as the last straw for an overstretched, vocationally-driven professional with closely aligned personal and professional identities. Occupational practitioners should feel empowered to advocate for such regulated workers. This professional support may provide an important safety mechanism to protect workers’ mental health. The depth of the distress caused by adverse regulatory scrutiny and judgement should not be underestimated; neither should the value of an occupational health clinician who can offer vital and nuanced guidance and support to those experiencing a time of work-related stress that can have potentially devastating consequences.

## About the authors

**Hester Mannion** is a specialist registrar in general and older adult psychiatry at East London Foundation Trust, London, UK. **Darren Minshall** is a medical officer and consultant psychiatrist at the Department of Community Mental Health, HM Naval Base Devonport, UK Defence Medical Services, Plymouth, UK. **Mark Tarn** is an Army GP and consultant psychiatrist in Occupational Psychiatry, Guy's and St Thomas’ NHS Foundation Trust, London, UK. **Derek K. Tracy** is Chief Medical Officer and consultant psychiatrist at West London NHS Trust, London, UK; and honorary senior lecturer at the Institute of Psychiatry, Psychology and Neuroscience, King's College London, London, UK. **Samantha K. Brooks** a post-doctoral research fellow at the Department of Psychological Medicine, King's College London, London, UK. **Neil Greenberg** is a professor of defence mental health and consultant psychiatrist at the Department of Psychological Medicine, King's College London, London, UK.
